# Short‐term insomnia and common cold: A cross‐sectional study

**DOI:** 10.1002/jgf2.278

**Published:** 2019-09-12

**Authors:** Simmon Gomi

**Affiliations:** ^1^ Gomi Family Clinic Suwa Japan

**Keywords:** common cold, cross‐sectional study, insomnia, Japan, mild short‐term insomnia

## Abstract

**Background:**

The role of insomnia as a symptom of cold has not been fully explored. This study aimed to identify the nature of mild short‐term insomnia (MSTI) as a symptom in common cold and examine the relationship between the diagnosis of common cold and MSTI.

**Methods:**

A cross‐sectional study was conducted at a clinic in Nagano, Japan. In this study, 32 participants were recruited as common cold patients, and 30 people without common cold were selected as the comparison group who did not have any symptoms of fever, cough, septum, rhinorrhea, or sore throat in this period.

**Results:**

About 75% of patients in the common cold group (CCG; 24 of 32) and 33% of total patients in the not common cold group (NCCG; 10 of 30) showed MSTI. The prevalence of MSTI was significantly more frequent in patients in CC (χ^2^ = 10.854, *df* = 1, *P* < .001). MSTI occurred on an average of 1.0 day (SD = 1.4) before the common cold onset and continued for a mean of 2 days (SD = 1.6). The frequency of fever was related to age, but MSTI appeared regardless of age.

**Conclusion:**

Mild short‐term insomnia is a common symptom in common cold.

## Abstract 2

Seventy‐five percent of the patients in the common cold (CC) group (n = 24 of 32) and 33% of those in the not common cold (NCC) group (n = 10 of 30) reported mild short‐term insomnia (MSTI). The proportion of patients reporting MSTI was significantly larger in the CC than NCC (χ^2^ = 10.85, *df* = 1, *P* < .001).

## Graphical abstract

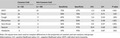

## 1 BACKGROUND

Patients with common cold who complain of short‐term insomnia are commonly seen in clinical practice; however, there has been no empirical research regarding the relationship between insomnia and common cold. If insomnia is a symptom of common cold, its presence may help to distinguish between common cold and similar illnesses and serious diseases. Thus, assessing for the presence of insomnia may be substantially beneficial for reducing diagnostic errors relative to using interviews alone. Further, identifying the presence of insomnia as a symptom of common cold would be beneficial for future research that attempts to differentiate severe diseases and illnesses similar to common cold as there has been no previous research on the relationship between insomnia and common cold.

There are two possibilities regarding the relationship between impaired overall health and insomnia: Insomnia contributes to the occurrence of impaired overall health, or impaired overall health leads to insomnia. Previous research has indicated that chronic insomnia is one of the causes of common cold or pneumonia.^1^, ^2^, ^3^, ^4^ According to previous studies,^5^ impaired overall health may affect insomnia. Research indicates that narcolepsy frequently accompanies influenza,^6^ suggesting the possibility that influenza influences sleep behaviors. However, the influence of short‐term health problems on insomnia in typically healthy populations has not been studied. Therefore, the presence and nature of insomnia due to short‐term impaired health are unknown. Common cold is a condition contributing to short‐term impaired overall health, and the nature of the relationship between insomnia and common cold has not been well examined.

Several studies have suggested the presence of factors that may confound the association between insomnia and poor health and illness. For example, existing research has studied insomnia due to symptoms such as rhinorrhea and cough. These studies of sleep quality and the common cold have explored the association between runny nose or nasal congestion and insomnia.^7^, ^8^ Similarly, cough and chronic obstructive pulmonary disease have been found to be related to insomnia.^9^ However, individuals may experience insomnia as a symptom of common cold, rather than as a cause of a cold, even when coughing and rhinorrhea are not serious. Thus, the role of insomnia as a symptom of cold is not well established. Based on existing research, both rhinorrhea and cough appear to be confounding factors in the relationship between common cold and the symptom of insomnia. No research to date has controlled for rhinorrhea and cough when examining the presence of insomnia as a symptom of common cold.

The reports on the prevalence of insomnia vary widely depending on the study design. The prevalence of insomnia with distress or impairment is 10%‐15%, and the prevalence of insomnia in the adult population is estimated at 33%‐55%.^10^ However, this reported insomnia may not represent the most recent insomnia, such as that on the day before the hospital visit, but are indicative of the frequency of insomnia existing for a prolonged period. For example, if one has experienced sleeplessness for a month, a few days may be counted as sleepless. The percentage of people who experience insomnia for several days prior to the common cold, which is necessary for comparison with the percentage of people with mild short‐term insomnia (MSTI), is unknown. The less biased method has been thought to be prevalence estimates based on ratings of both MSTI and insomnia on the same questionnaire, which was the method selected for the current study.

Insomnia may be useful for diagnosing a patient with common cold if it is identified as a symptom of a cold. Thus, this study also investigated the possibility that insomnia would be a useful symptom when diagnosing common cold. The goal of this study was to identify the nature of MSTI as a symptom in common cold and clarify its diagnostic characteristics.

## 2 MATERIALS AND METHODS

This cross‐sectional study was conducted at a nonbed clinic in Nagano, Japan, between October 29 and December 2, 2018. Patients were clearly informed that there is a possibility of using their data for research purposes. Patients were told that they could refuse to participate in the research if they so desired.

### 2.1 Participants

**Figure 1:**
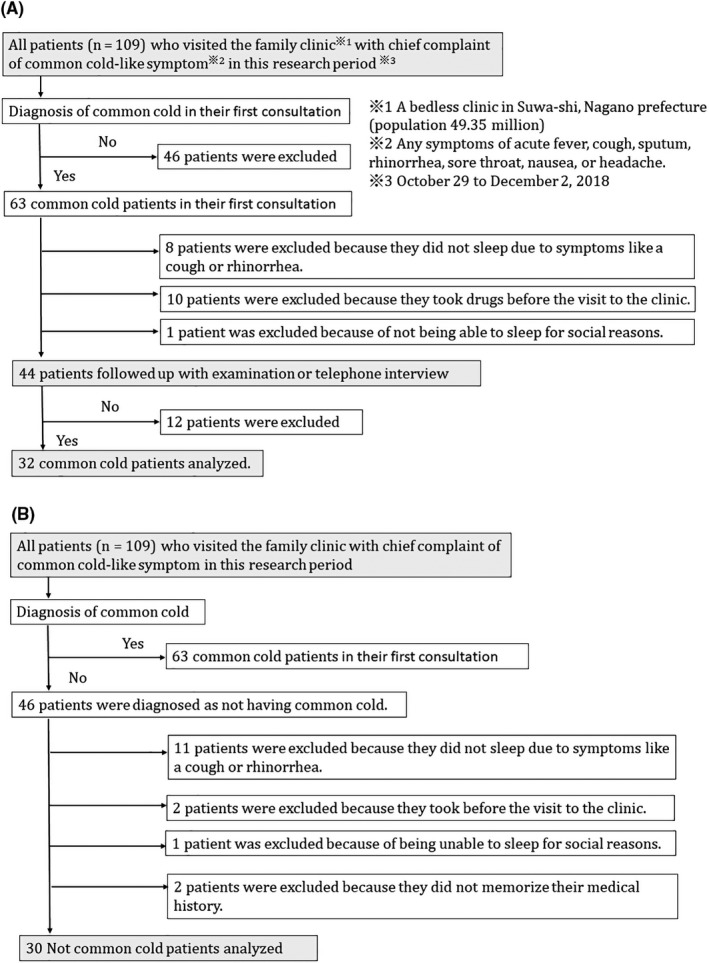
Study Flowchart. (A) indicates study flowchart of common cold group, and (B) indicates one of the not common cold groups

All patients of the clinic with any acute symptom of fever, cough, rhinorrhea, headache, or sore throat during this time period were invited to participate in this study. When a physician determined that there was a possibility of insomnia, the patient was rated as being positive for the symptom of insomnia. Patients completed the checklist prior to the diagnosis, and no items from the symptom list were added to the checklist after the patient was diagnosed by the physician. The common cold group (CC) comprised patients who were clinically diagnosed by me as having common cold during their first consultation, and who also participated in a follow‐up examination or telephone interview to confirm the occurrence of spontaneous remission. The patients in this group had diagnoses equivalent to J00 (acute nasopharyngitis) in the ICD‐10. The not common cold group (NCC) included those who were not diagnosed with common cold in their first consultation (Figure 1).

I excluded all patients who were unable to ascertain that they had spontaneous remission (Figure 1). Further, people who took over‐the‐counter cold medicine the day they became aware of symptoms were excluded as these cold medicines often contain ingredients that affect sleeping, such as anticholinergic drugs. Patients who had difficulties sleeping due to personal reasons, such as those who could not sleep due to working the night shift, were excluded because they were judged to be unable to have an accurate sleep evaluation. Based on existing research, I considered the latent period of the cold as being 24 to 72 hours,^11^ which meant that insomnia would not be identified as the cause of a common cold if it appeared on the same day as the onset of the cold or on the day before the onset of the illness. Common cold includes multiple symptoms such as rhinorrhea, cough, and sore throat.^6^ Patients with only one symptom (eg, rhinorrhea only, cough only, sore throat only) were excluded because diseases other than a common cold may be present if there is only one symptom. To diagnose a common cold, it is essential that the common cold resolves through spontaneous remission.^12^ Finally, patients who did not follow up after recovering from their illness at least within one month after consultation were excluded from the final analytic sample.

### 2.2 Procedures

A medical questionnaire was given to all patients, and their symptoms were confirmed through a diagnostic interview with a physician. Based on this interview sheet, details on the period of illness and the nature of all symptoms were obtained. The questionnaire included a checklist with the following symptoms: fever, sore throat, cough, sputum, runny nose, joint pain, headache, abdominal pain, back pain, nausea, vomiting, weight loss, weight gain, diarrhea, constipation, loss of appetite, cold, shivering, trouble breathing, fatigue, dizziness, and insomnia. For each question, there were a checkbox and a free‐response field.

In order to check the status of insomnia several days before the hospital visit, I investigated the total number of visits for influenza vaccination by patients visiting the same clinic between November 24 and December 1, 2018 (seventh day of medical treatment). There were 56 people, including 22 men, with an average age of 37.8 years (SD 27.1). The same questionnaire was given to all patients in the CC and the NCC groups. Specifically, I asked patients who came to the hospital for an influenza vaccination about their last experience of sleeplessness before the hospital visit for comparison to those patients in the CC and NCC groups.

### 2.3 Statistical analyses

Chi‐square tests were used to assess differences in gender and age groups. The Mann‐Whitney U test was used to evaluate the difference between the two groups with regard to age. Although it was difficult to estimate the sample size because there was no previous research, MSTI was regarded as the primary explanatory variable of common cold, while cough and rhinorrhea were identified as confounding factors based on previous research. In order to use these three explanatory variables, the sample size of both the CC and NCC groups was required to be 30 or more based on the minimum 10 events per variable (EPV) rule.^13^ This study satisfied the EPV rule. A significance level of *P* < .05 was used in the analyses. The relationship between MSTI and other symptoms was analyzed using logistic regression analysis. All statistical analyses were performed using R 3.5.2.^14^

### 2.4 Ethical considerations

All data and information provided by participating patients were anonymous. The questions that were given to all patients included a reference to the research that was being conducted and that the questionnaire was being used for this purpose. The completion and return of the questionnaire was considered to indicate consent. All participants were told that they could refuse to participate at any point in time and that there would be no disadvantages or repercussions for their refusal to participate. Additionally, the questionnaire used in the clinic during the initial consultation was used in this study as well. The questionnaire used in this study is one that is commonly used during patients’ first visit to this hospital, and no special examination was performed beyond the routine examination by the physician for study participants. Further, I analyzed the data after the patients’ diagnosis and treatment to reduce any risks or potential harm to the patients, and no aspects of their treatment were affected by their inclusion in the study. Finally, as research data that contained personal information were not included in the study, no personal information was extracted from the hospital records. This clinical research was carried out in accordance with the 1964 Declaration of Helsinki. The research protocol for this study was approved by the Japan Primary Care Association's Ethics Committee (approval number: approved number: 2019‐002).

## 3 RESULTS

The total sample included 32 patients in the CC (10 men; age range 0.5‐84 years) and 30 in the NCC (14 men; age range 0.5‐82 years).

Table 1 shows that the patients’ clinical and sociodemographic characteristics were homogenous, given that there were no statistically significant differences in age and gender between the two groups (*P* > .05). The NCC’s final diagnoses included gastroenteritis (n = 11), sinusitis (n = 3), allergic rhinitis (n = 3), fever only (n = 3), asthma (n = 2), bronchitis (n = 1), pneumonia (n = 1), atopic cough (n = 1), urticaria (n = 1), varicella (n = 1), migraine (n = 1), pyelonephritis (n = 1), and myalgia (n = 1). During the study period, no influenza patients visited the clinic.

**Table 1:** Patients’ backgrounds and comparison between common cold and not‐common cold patients

	Common cold	Not‐common cold	*P* value
	(n = 32)	(n = 30)	
Male, n (%)	10 (34)	14 (47)	.30
Age (years), Median (IQR)	31.9 (18.5)	30.2 (19)	.75
Age group (years), n (%)			
≤9	10 (31)	12 (40)	.83
10‐19	6 (19)	3 (10)	.51
20‐49	6 (19)	6 (20)	>.99
50‐69	5 (16)	5 (17)	>.99
≥70	5 (16)	4 (13)	>.99

Note

Chi‐square tests were used for gender and age groups. Mann‐Whitney U test was used for age.

Abbreviation: IQR, interquartile range.

**Figure 2:**
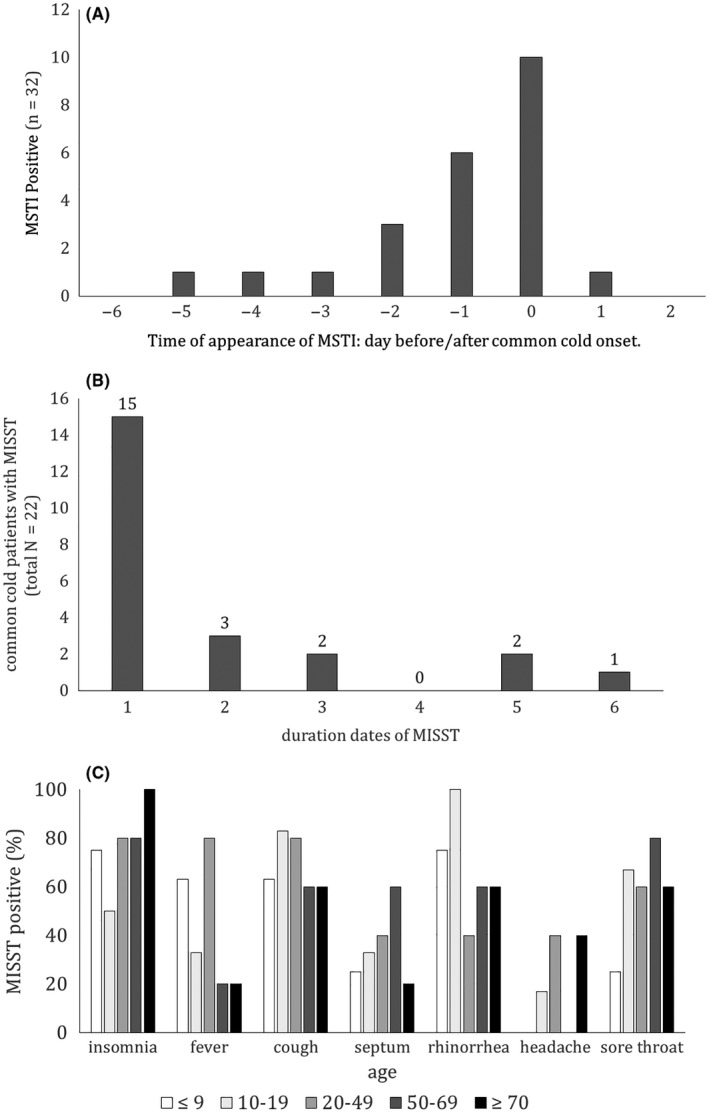
Features of Mild Short‐Term Insomnia (MSTI). In order to show the clinical nature of MSTI, it is necessary to show when, for how long, and for whom MSTI occurs; hence, the figure shows (A) emergence times, (B) duration of MSTI, and (C) MSTI positivity by age

Seventy‐five percent of the patients in the CC (24 of 32) and 33% of those in the NCC (10 of 30) reported MSTI. The proportion of patients reporting MSTI was significantly larger in the CC than NCC (χ^2^ = 10.854, *df* = 1, *P* < .001; Table 2). Logistic regression analysis revealed that CC was significantly associated with the presence of MSTI, cough, and rhinorrhea (Table 3). MSTI occurred on an average of 1.0 day (SD = 1.4) before the onset of common cold and continued for an average of 2.0 days after its onset (SD = 1.6; Figure 2).

**Table 2:** Sensitivity, specificity, and likelihood ratios for each symptom

	Common Cold	Not‐Common Cold	Sensitivity	Specificity	LH+	LH−	*P* value
	(n = 32)	(n = 30)					
MSTI	24	10	75%	67%	2.3	0.38	<.001
Fever	16	9	50%	70%	1.7	0.71	.110
Cough	22	9	69%	73%	2.6	0.43	.002
Rhinorrhea	22	7	69%	83%	4.1	0.38	<.001
Sore throat	18	2	56%	93%	8.4	0.47	<.001
Sputum	11	1	34%	97%	10.0	0.68	.002
Headache	6	2	19%	93%	2.8	0.87	.016

Note

Chi‐square tests were used to compare differences in the proportion of common and not‐common cold groups.

Abbreviations: LH+, positive likelihood ratios; LH−, negative likelihood ratios; MSTI, mild short‐term insomnia.

**Table 3:** Odds ratios for common cold by mild short‐term insomnia, cough, and rhinorrhea

	Odds ratio	95% confidence interval	*P*‐value
Insomnia	16.0	2.72‐94.00	.002
Cough	9.14	1.94‐43.00	.005
Rhinorrhea	24.3	3.99‐148.00	<.001

Note

Odds ratios and confidence intervals were calculated based on Wald's method.

Sixty‐three participants were diagnosed with a cold, including those who were ultimately excluded from the analyses, indicating a sensitivity of 65% and a specificity of 51% (χ^2^ = 8.26997, *df* = 1, *P* = .004) of being correctly categorized in the CC. Patients in the CC who received medication prior to visiting the clinic had an MSTI positive rate of 33%, with *P* = .047 compared to those in the NCC group. There was no gender difference in the incidence of MSTI (Table 1).

Forty‐nine percent of patients aged under 49 years (21 of 43) and 4% of those aged over 50 years (4 of 19) had fever. The proportion of patients with fever was significantly larger in patients under 49 years (χ^2^ = 5.34, *df* = 1, *P* = .02).

An investigation of the total number of visits for influenza vaccination by patients visiting the same clinic between November 24 and December 1, 2018 (seventh day of medical treatment), revealed that the rate of chronic insomnia was 9% (5 out of 56), and the rate of MSTI without chronic insomnia was 0% (0 out of 56, *P* < .050), indicating that MSTI is an acute symptom associated with a cold.

## 4 DISCUSSION

Our findings demonstrated that MSTI was frequently found in patients diagnosed with common cold (Table 2). Even after adjusting for the symptoms of cough and rhinorrhea, MSTI continued to be significantly associated with a diagnosis of common cold. Insomnia was an important factor for common cold. In many cases, MSTI appeared as trivial symptoms. Even if MSTI is the result of a co‐occurring symptom, its presence can be useful for the diagnosis of common cold. The frequency of symptoms was higher in MSTI than in fever. As age increases, the incidence of fever decreases; however, the presence of MSTI does not decrease (Figure 2C). Of note, MSTI seems to be more useful than fever for diagnosing common cold in people of all ages.

The high prevalence of insomnia has raised concerns about the possibility of a high misdiagnosis rate; however, according to research conducted with the influenza vaccination group, insomnia rarely appears several days before the specific day that was identified as the onset of the illness, and MSTI's diagnostic value for common cold is significant. This indicates that the diagnostic value of MSTI is not negligible, despite the prevalence of general insomnia, and its influences merit empirical consideration.

People with gastroenteritis in the NCC had the highest rate of MSTI (50%; 12 out of 24 people). From this, it can be inferred that MSTI is indicative of some general state of discomfort or malady. Further, MSTI might be useful as a means of distinguishing between CC and NCC for patients who have had cold‐like symptoms. It may be helpful to diagnose patients with conditions other than cold, who will be visiting with symptoms like that of cold, such as pneumonia and sinusitis.

Elderly people may be more likely to report potential insomnia than are younger adults.^15^ However, our present sample size was too small to consider the role of potential sleeplessness in the elderly and its relationship with MSTI. Four patients in the CC reported chronic insomnia, and two of them were aged over 70 years.

Thirteen patients reported that they had taken an over‐the‐counter multisymptom cold medicine on the first day of experiencing symptoms of cold, and therefore were excluded from the final analysis because the medications contain ingredients affecting sleep patterns, such as antihistamines. Thirty‐eight percent of these patients (5 of 13) were MSTI positive. There was a significant difference between the 13 patients who were excluded and the other 32 CC (χ^2^ = 5.39, *df* = 1, *P* = .02), indicating that multisymptom cold medicine containing antihistamines appeared to reduce MSTI. However, their consistent use is not recommended because MSTI is an extremely time‐limited symptom of common cold, and hence, using a multisymptom cold medicine for several days is excessive.

Although this study cannot draw any inferences regarding causality due to the cross‐sectional nature of our data collection, it can demonstrate that not all MSTI lead to a common cold because most patients had MSTI on the night of the cold onset. If MSTI was the cause of common cold, common cold would occur after MSTI. If common cold occurs after the infection has been identified by the presence of insomnia, the serial order of the occurrence of symptoms will be insomnia, the establishment of infection, and the onset of illness. In other words, the time from insomnia to the onset of common cold should be longer than the incubation period. The number of days from insomnia to the onset date is shorter than that of the onset date from the incubation period. Many patients developed insomnia after the onset of common cold. Considering the incubation period of the common cold, MSTI appears to not be the cause of a cold, but rather the result of the cold.

Using a follow‐up telephone interview, I was able to confirm the duration of MSTI and remission of symptoms. In order to diagnose a common cold, a natural remission is necessary. However, in the study, there were no cases where the diagnosis of a common cold changed after the follow‐up.

The pathophysiology of MSTI and the confounding factors other than cough and rhinorrhea merit further empirical study. Further, the relationship between influenza and MSTI is a subject for future research.

Several limitations are worth noting in this study. First, reporter bias could have occurred in the data collection method, as patients are asked directly about these symptoms, and some may believe that some symptoms, such as insomnia, are irrelevant. As there was only one researcher, it was not possible to establish interrater reliability for the diagnostic categorization, which should be addressed in future studies. The pattern of results may be different in case of an epidemic of a cold virus, so future continuous follow‐up is necessary. Finally, as this was a cross‐sectional design, no causal association between insomnia and common cold could be determined.

## 5 CONCLUSION

Mild short‐term insomnia is a common symptom seen in patients diagnosed with common cold. MSTI may affect methods of distinguishing common cold and related diseases. It may also affect insomnia treatment.

## CONFLICT OF INTEREST

I have stated explicitly that there are no conflicts of interest in connection with this article.
